# Supplementary material for: CXCL12 Neutralizing Antibody Promotes Hair Growth in Androgenic Alopecia and Alopecia Areata
Source: Int J Mol Sci. 2024 Jan 30;25(3):1705. doi: 10.3390/ijms25031705 (PMC10855715; doi:10.3390/ijms25031705)
Supplement: Supplementary file 1 [file ijms-25-01705-s001.zip › ijms-2810769-supplementary.pdf]

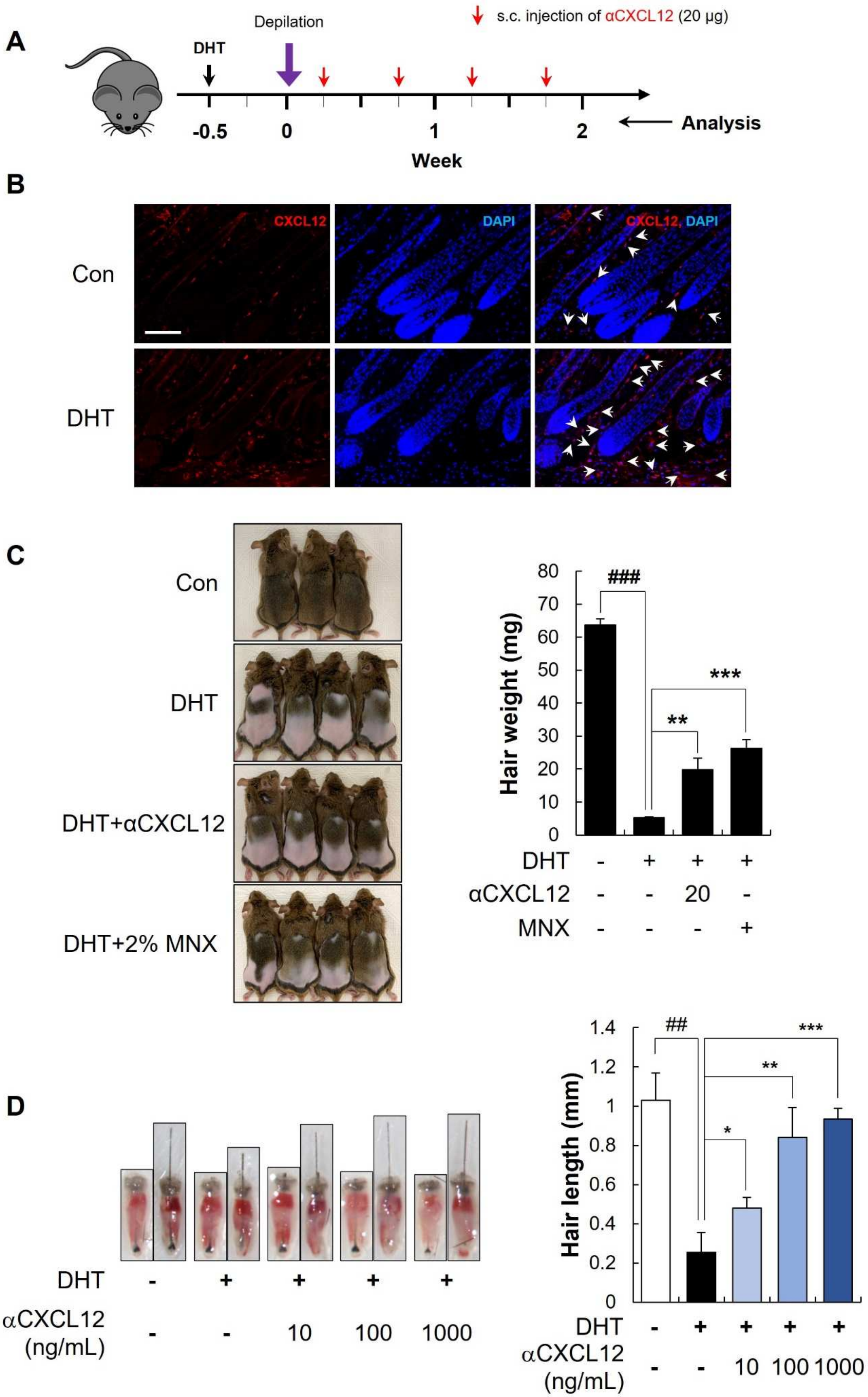
A
s.c. injection of αCXCL12 (20 μg)
Depilation
DHT
-0.5
0
1
2
Week
Analysis
B
CXCL12
DAPI
CXCL12, DAPI
Con
DHT
C
Con
DHT
DHT+αCXCL12
DHT+2% MNX
Hair weight (mg)
80
70
60
50
40
30
20
10
0
###
**
***
DHT - + + +
αCXCL12 - - 20 -
MNX - - - +
D
DHT - + + + +
αCXCL12 (ng/mL) - - 10 100 1000
Hair length (mm)
1.4
1.2
1
0.8
0.6
0.4
0.2
0
##
*
**
***
DHT - + + + +
αCXCL12 (ng/mL) - - 10 100 1000

Figure S1. Neutralization of CXCL12 promotes hair growth in a DHT-induced AGA model. (A) The back skin of 7-week-old C_3H male mice was shaved, and subcutaneous injections of DHT were used to induce the AGA model. (B) The expression of CXCL12 in the dorsal skin of DHT-treated mice increased. $CXCL12^+$ cells (red) are indicated by white arrows, and DAPI staining (blue) indicates cell nuclei. The scale bar is set at 100 μm. (C) αCXCL12 (20 μg per head) were subcutaneously injected in the presence of DHT twice a week for 2 weeks. A daily topical treatment of 2% minoxidil served as a positive control. αCXCL12 administration accelerated hair growth in DHT-treated C_3H mice, and the hair weight was measured. ### $p < 0.001$ vs control, ** $p < 0.01$, *** $p < 0.001$ vs DHT-treated. (D) αCXCL12 treatment increased the length of mouse vibrissa follicles in the AGA mimic ex vivo model. αCXCL12 (10, 100, or 1000 ng/mL) were treated in the presence of 100 nM of DHT for 48 h. ## $p < 0.01$ vs control, * $p < 0.05$, ** $p < 0.01$, *** $p < 0.001$ vs DHT-treated. The asterisk and sharp symbols indicate statistical differences in the Student's *t*-test.

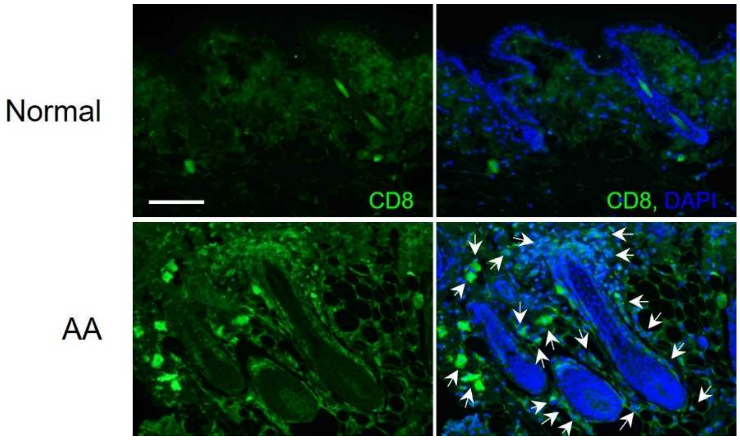

Figure S2. Expression of CD8^{+} cells in alopecia areata model. SDLN cells were isolated from AA mice and intradermally injected into the dorsal skin to induce AA. Severe hair loss was observed after 12 weeks, and skin sections from normal and AA mice were stained with an anti-CD8 antibody (green). CD8^{+} cells were increased in AA model. DAPI staining (blue) indicates cell nuclei. The scale bar is set at 100 μm.

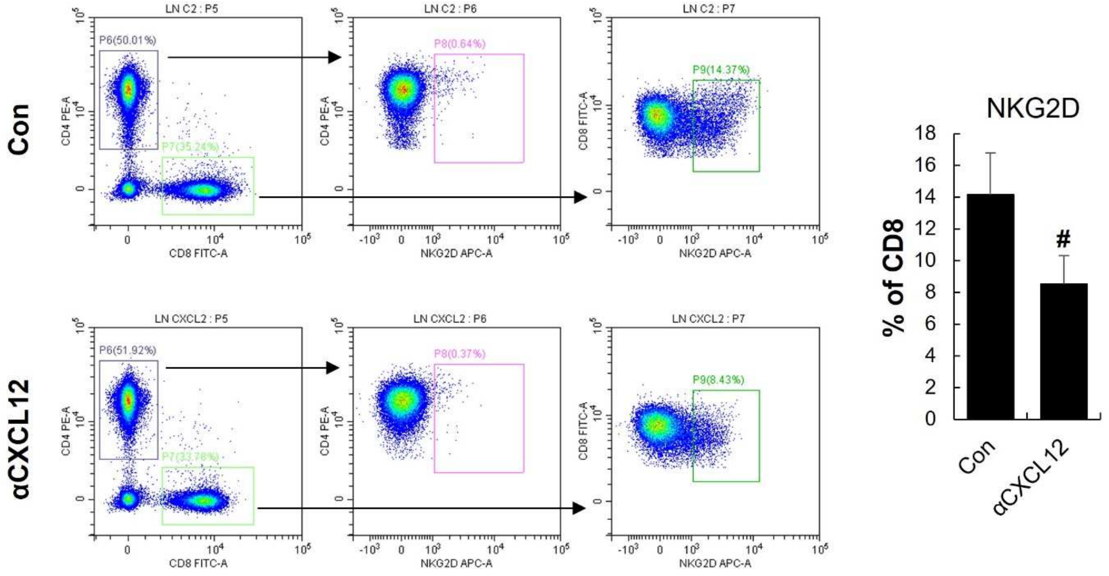

Figure S3. CXCL12 neutralization decreased the expression of NKG2D$^+$ cells in CD8$^+$ T cells. Flow cytometric analysis was performed to measure the NKG2D expression in CD8$^+$ T cells by gating SDLN cells from two groups, and αCXCL12 treatment reduced the NKG2D expression. # $p < 0.05$, vs control. The sharp symbols indicate statistical differences in the Student's *t*-test.

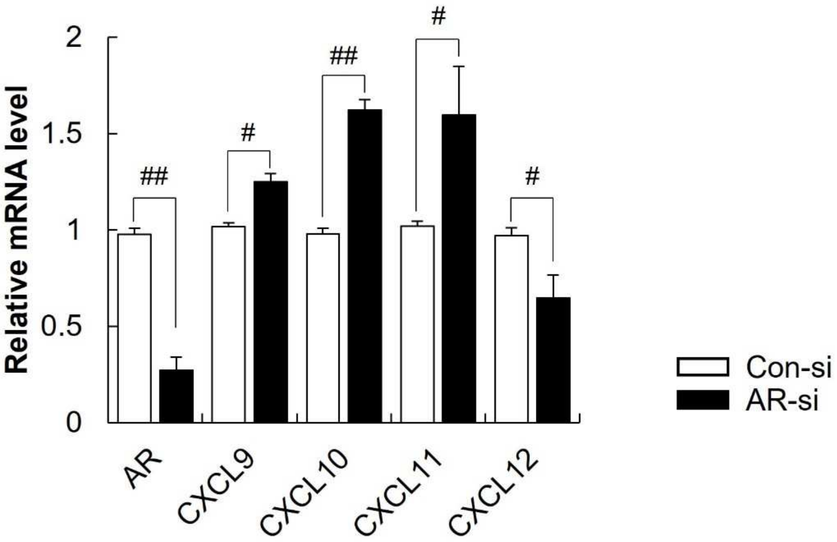

Figure S4. mRNA expression of CXCL families after transfection of androgen receptor siRNA. Knockdown of androgen receptor (AR) significantly down-regulated CXCL12 mRNA, while increased CXCL9, CXCL10, and CXCL11 in dermal fibroblasts. # $p<0.05$, ## $p<0.01$ vs. control. A sharp indicates a statistical difference in a Student's *t*-test.

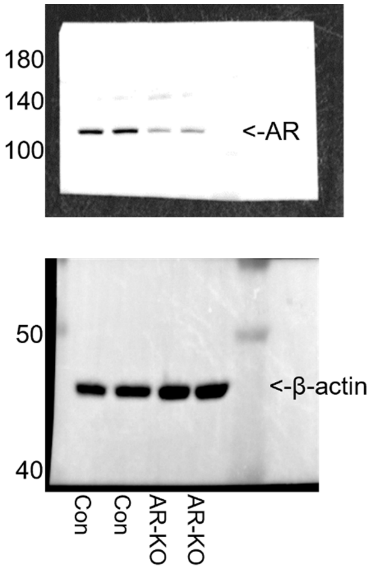

Original western blot for Figure 2D.

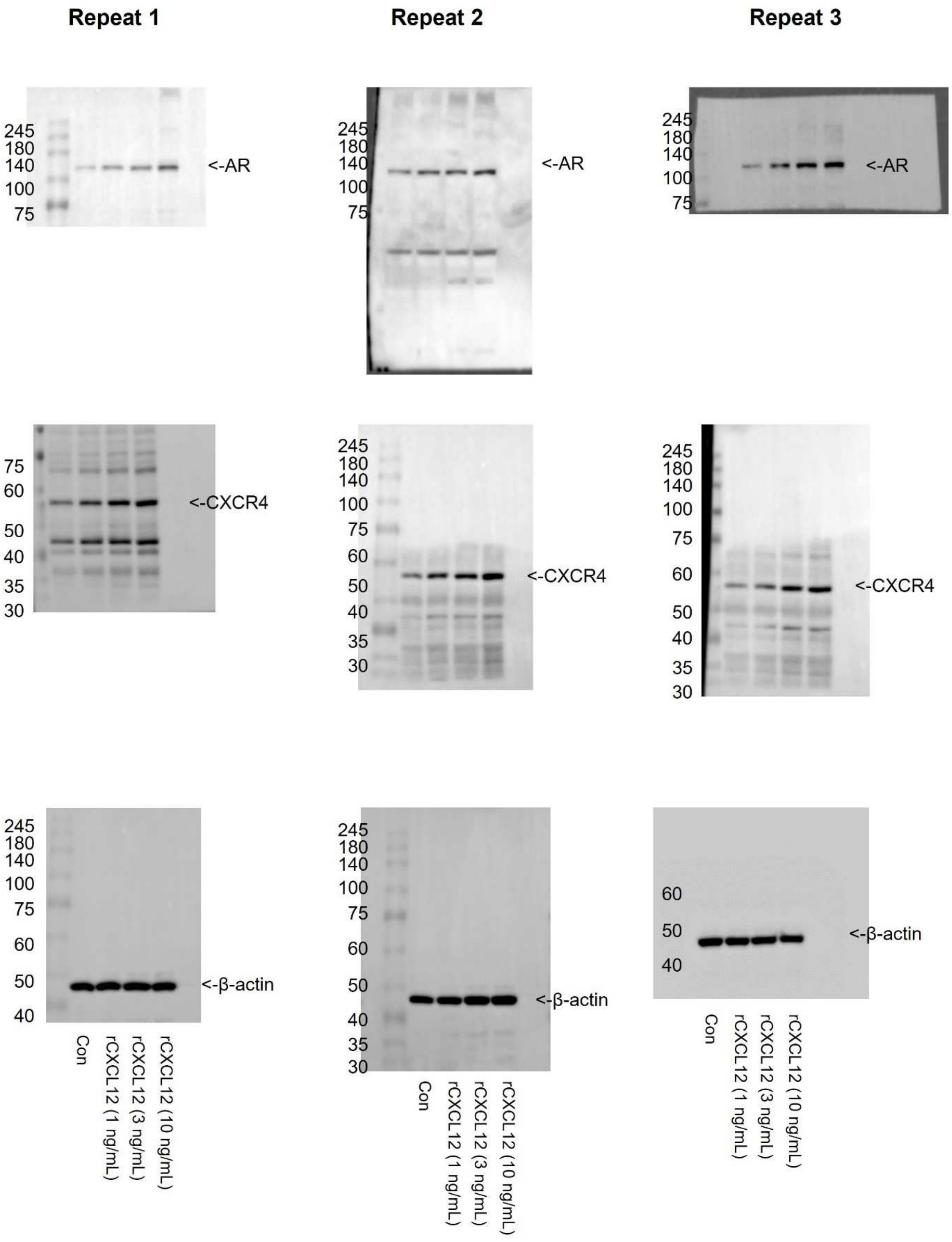

Original western blot for three repeats (Fig.3C)
